# Perspectives on returning individual and aggregate genomic research results to study participants and communities in Kenya: a qualitative study

**DOI:** 10.1186/s12910-022-00767-y

**Published:** 2022-03-18

**Authors:** Isaac Kisiangani, Shukri F. Mohamed, Catherine Kyobutungi, Paulina Tindana, Anita Ghansah, Michele Ramsay, Gershim Asiki

**Affiliations:** 1 African Population and Health Research Center (APHRC), P.O. Box 10787, Nairobi, 00100 Kenya; 2 School of Public Health, College of Health Sciences, University of Ghana, Legon, Ghana; 3 Noguchi Memorial Institute for Medical Research, College of Health Sciences, University of Ghana, Legon, Ghana; 4 Sydney Brenner Institute for Molecular Bioscience, The University of Witwatersrand, The Mount, 9 Jubilee Rd, Parktown, Johannesburg, 2193 South Africa

**Keywords:** Genomics, Return of results, Aggregate level, Individual level

## Abstract

**Background:**

A fundamental ethical challenge in conducting genomics research is the question of what and how individual level genetic findings and aggregate genomic results should be conveyed to research participants and communities. This is within the context of minimal guidance, policies, and experiences, particularly in Africa. The aim of this study was to explore the perspectives of key stakeholders' on returning genomics research results to participants in Kenya.

**Methods:**

This qualitative study involved focus group discussions (FGDs) and in-depth interviews (IDIs) with 69 stakeholders. The purposively selected participants, included research ethics committee (REC) members (8), community members (44), community resource persons (8), and researchers (9). A semi-structured interview guide was used to facilitate discussions. Six FGDs and twenty-five (IDIs) were conducted among the different stakeholders. The issues explored in the interviews included: (1) views on returning results, (2) kind of results to be returned, (3) value of returning results to participants, and (4) challenges anticipated in returning results to participants and communities. The interviews were audio-recorded, transcribed verbatim, and coded in Nvivo 12 pro. Thematic and content analysis was conducted.

**Results:**

Participants agreed on the importance of returning genomic results either as individual or aggregate results. The most cited reasons for returning of genomic results included recognizing participants' contribution to research, encouraging participation in future research, and increasing the awareness of scientific progress. Other aspects on how genomic research results should be shared included sharing easy to understand results in the shortest time possible and maintaining confidentiality when sharing sensitive results.

**Conclusions:**

This study identified key stakeholders’ perspectives on returning genomic results at the individual and community levels in two urban informal settlements of Nairobi. The majority of the participants expect to receive feedback about their genomic results, and it is an obligation for researchers to see how to best fulfil it.

## Background

Return of results to study participants and communities involved in research has become a major topic of debate [1–4]. The issue stems from the gap between the preferences favouring returning research results that many study participants and community members want versus the past research practice to avoid returning results to the research participants [5]. The controversy is whether or not researchers have any responsibility to offer participants results [6–10]. Other questions remain about what results should be communicated to individuals and communities, how to communicate the results, and who should be involved in the process [11]. Researchers' main concern is integrity and the societal benefit of research rather than the participants' best interests [12]. They maintain that returning results could cause undue distress whereby the risks are likely to outweigh potential benefits [13–15]. Despite this, there are increasing calls to return research results to study participants aligned with participant engagement and open science [16–20]. The consensus is that returning research results to participants is good practice, based on the moral obligation of respect for persons [11, 21–23] and on a ‘soft’ duty of care owed to research participants, particularly when the findings are clinically significant [24].

In genetic and genomic research, the focus has primarily been on what information, if any, to offer back to research participants considering the inconclusiveness surrounding most genetic results [25–27]. Some authors argue that sharing aggregate findings from genomic studies should be viewed as ‘sharing knowledge’ rather than ‘returning results’ [28]. The impact of genomic research will be enhanced when communities embrace the results and fully understand its value to their families and future generations [29, 30]. If appropriately communicated, the results are likely to foster a sense of uniqueness in the context of a larger population.

There is very little evidence on how to involve communities in genomic research beyond the sampling and data collection stages, which includes feedback of findings [31]. This also includes modalities of disseminating individual and aggregate genomic research results to study participants and their communities [32, 33]. The dissemination of aggregate findings as part of knowledge sharing not only accurately describes the exercise, but also helps to alleviate some of the risks associated with returning non-individual aggregate results’ [28]. One of the biggest challenges in sharing aggregate knowledge is determining appropriate methods and how communities’ perspectives should be incorporated into policies guiding when, how, and what to return to study participants and communities involved in genomic research. The Human Heredity and Health in Africa Initiative (H3Africa) developed and released guidelines in 2018 to assist genomics researchers in deciding what and when to share individual genetic findings [34]. These guidelines have become important in sub-Saharan African countries because of the lack of national guidance on genetic research [35].

This study sought to address this gap by exploring views from key stakeholders and community members on how genomic results should be communicated to study participants and communities. The evidence generated in this study will be useful in informing future research on disseminating findings to the community.

## Methods

The study is reported per a set of standardized criteria for reporting qualitative research (COREQ) [36].

### Study site

The study was conducted in two Nairobi urban informal settlements in Kenya (Korogocho and Viwandani) whose populations have been under demographic surveillance since 2002 as part of the Nairobi Urban Health and Demographic Surveillance System (NUHDSS) [37, 38].

### Study design

Focus group discussions (FGDs) and in-depth interviews (IDIs) were used in the study as part of an exploratory qualitative design with a participatory approach. We used purposive sampling procedures to identify and conveniently select various categories of stakeholders. Four stakeholder groups were formed. Stakeholder group 1 included community members selected from a genomic study (African Wits-International Network for Demographic Evaluation of Population and Their Health (INDEPTH) partnership for Genomic Studies (AWI-Gen) phase I study participants (n = 44). The AWI-Gen phase I study’s main goal was to identify the contribution of genetic and environmental factors to cardiometabolic disease in Africans [39]. Details of the AWI-Gen study are described elsewhere [40]. Stakeholder group 2 consisted of community resource persons, including village elders (n = 2), administrators (n = 2), community health volunteers (n = 2), and religious leaders (n = 2). Stakeholder group 3 consisted of the research team of AWI-Gen phase I in Nairobi, i.e., the genomic researchers (n = 2), fieldworkers (n = 4), study coordinator (n = 1), and laboratory technologists (n = 2). Stakeholder group 4 were members of research ethics committees who reviewed genomic study protocols in Kenya (n = 8). All participants were invited to participate in the interviews either by email or in person by trained research assistants. We contacted a total of 80 participants but 11 declined to participate due to various reasons including "lack of confidentiality" and "busy schedules." Table 1 contains a summary of all participants.

**Table 1:** Distribution of participants

Data collection type	Participants	No. of interviews	Total participants
Focus group discussions (FGDs)	AWI-Gen I women	2	15
	AWI-Gen I Men	2	16
	REC members	1	7
	AWI-Gen I research team	1	6
In-depth interviews (IDIs)	AWI-Gen I community members	13	13
	AWI-Gen I researchers	2	2
	Administrators	2	2
	Village elders	2	2
	Religious leaders	2	2
	AWI-Gen I field coordinator	1	1
	REC member	1	1
	Community Health Volunteers	2	2
Total		31	69

### Research team

One of the seven authors (I.K.; S.F.M.; C.K.; P.T.; A.G.; M.R.; and C.K.) with a professional interest in genetics and community engagement conducted some interviews. All the researchers have worked in the medical field and have experience conducting in-depth interviews. Before the interviews, the interviewers had no prior knowledge of the study participants, either personally or professionally.

### Research instruments

The initial interview guides were based on questions that the researchers had identified after reviewing the literature, and the guides were updated as new themes emerged throughout the study. Stakeholder groups 1–4 were interviewed using an interview guide containing organized and unstructured questions. FGDs with groups 1, 3, and 4 used a similar semi-structured discussion guide to elicit in-depth perspectives and discussion on returning results to study participants.

### Data collection

We collected data between October 2019 and January 2020. Data were collected by trained interviewers using semi-structured interview guides. The interviews were conducted in both English and Kiswahili. The team was trained on the study rationale, the objectives, research strategies, data collection procedures, note-taking during the interviews, research ethics and how to get informed consent. The interviews lasted 45–60 min and the conversation were allowed to flow naturally.

IDIs and FGDs were held in a private setting selected by the participants, most usually in their homes or workplace. A total of 6 FGDs and 25 IDIs were held. There were 6–10 participants in each of the FGDs. Table 1 shows the different types of interviews conducted and the distribution of study participants by the type of interview conducted. We used a piloted interview guide in each study site to obtain information mainly on: (1) opinions on returning results; (2) types of results to be returned; (3) value of returning results to participants and (4) challenges to be faced in returning research results to participants and communities.

### Data management and analysis

The audio files were first transcribed verbatim then the Kiswahili transcripts were translated into English. The notes taken during the interviews were used to complement the transcription. The transcripts were labelled with the location of the interviews, the date of the conversation, the gender of the group, and the role of the group in the community. We read the transcripts and developed themes from the participant data and research questions to form a coding frame. We used QSR International’s Nvivo 12 software (QRS International Pty Ltd, 2014) to code the data [41]. Two research team members reviewed and coded the transcripts to ensure objectivity and consistency of the coded information. Following the coding of the transcripts, the two research team members looked for patterns in data and made connections to pre-existing and emerging themes. They compared the outcomes to see if there was any agreement. A discussion was held in case there were disagreements until they reached a consensus. The following themes were pre-established: taking feedback of research results seriously, the relevance of information, emotional reaction, types of results, methods of returning results, timing of the results, benefits, effects, understanding results, good ethical practices, challenges, and strategies to address the challenges.

Analysing the data from different perspectives strengthened external validity. We triangulated interview data from various sources (community members, study researchers, and research ethics committee members) to improve the internal validity of this study [42]. At the analysis stage, data saturation was attained when no new themes were identified.

## Results

### Respondent characteristics

The demographic characteristics of respondents are presented in Table 2. In total, 69 participants participated in the study. The majority of the participants were Christians with a fairly equal gender representation.

**Table 2:** Demographic characteristics

Participants	Research staff	Community member: Awigen I participants	CHVs	Administrators	Religious leaders	Village elders	REC members	Staff-field workers
N = 69	2	44	2	2	2	2	8	7
Age range in years	35–44	45–68	48–50	33–56	44–60	70–72	28–71	26–38
Median age (years)	40	53	49	45	52	71	40	32
Mean age (years)	40	54	49	45	52	71	47	32
Men	2	19	1	2	2	2	5	3
Women	0	27	1	0	0	0	3	4
Education	Tertiary (2)	None (1)	Primary incomplete (2)	Secondary (1)	Secondary (1)	Primary incomplete(2)	Tertiary (8)	Secondary (2)
		Primary incomplete (20)		Tertiary (1)	Tertiary (1)			Tertiary (5)
		Primary complete (8)						
		Secondary (14)						
		Tertiary (1)						

### Importance of genomic research results

Study participants perceived the return of results to be an important aspect of genomic studies. They noted that the return of results enabled them to know their health status and seek appropriate care.

It is good to return the results to the community. This is because when they say the number of those with high blood pressure against those who don’t, those with diabetes compared to those without, it means those who don’t have will be determined to go for the tests so that they can know their status whether they have high blood pressure or diabetes. If you keep silent, the locals in Korogocho cannot know the problems they have. **FGD Women, Korogocho, R6.**

The majority of study participants felt that returning results is a sign of respect to them and it maintains the relationship between them and researchers.

First of all, it builds trust; trusting one another because I got to know that the research that you are doing. One, because [Researchers] continue to do as [they] say. Thus, the first thing it [did was making] I trust you. Second, [I would like] to state that the results confirmed what I had already tested. Even though I knew [my condition], the results [confirmed it]. I knew you had done the tests and diagnosed that problem. **FGD Males, Viwandani, R10**.

Despite the researchers’ efforts to return results to study participants, some participants reported not receiving their results. They encourage the research team to make sure results are returned to all study participants.

They did not give us feedback. Therefore, [Researchers] should give us feedback on the research they carried out before. They should tell us, "Yes, we did not diagnose anyone with complicated diseases." Or, “We found out that a few people have complicated diseases and we will do this and this so that the disease does not spread or affect young children. **FGD Women, Viwandani, R4**.

The return of results to study participants influences their future participation in research studies. Majority of the study participants noted that they would be more motivated to participate in future studies if the researchers returned the results from the earlier study.

The current method used to return results can influence someone not to participate in the research because they are not sure if they will get results or not. It is now years since they did blood tests and we are just wondering if ever we will receive our results. Therefore, even if I am told that there is another research being done, I will not be motivated to participate because I am unsure if I will receive the results. It would be good if the results were [made available] in real-time or shortly afterward to motivate more people [to participate] in the research. They should have a good plan of returning results to motivate people to participate. **FGD Males, Korogocho, R1**.

Where the return of results is not done, it will harm future studies as people will not be willing to participate.

It will have a problem because the next time when you will need to come to carry out more research, it helped you at first while taking the samples but if you come back to the community and they haven’t known how the first one went. It will be [similar] to [a situation] in school where learners [are] given homework [but] the teacher does not mark it, [so] the learners do not know whether they passed or not. The teacher [then assigns] them another assignment [without telling] them how the first one went. So, in a community, if people have not been given the results of [previous] research, it will be difficult to give another sample for research because they will [inquire] about the first one [for which] they [have not yet received] the results yet and question the possibility of receiving the results of the current one. **IDI Community Leader 1, Korogocho**.

### Relevance of information for self and others

The majority of the AWI-Gen participants believe that receiving feedback impacts their health. Some felt it was unimportant because they might already have the disease when returning the results. They believe that delayed results mean that they will not effectively track the disease progression and make informed decisions around lifestyle adjustments.

I believe the results should be returned so that the community [can] understand what is going on and individuals [can] understand their status. Because, even during that program we only [learned about] the superficial conditions [and] did not receive blood test results, and we would like to [learn more about] what is happening in our bodies. **FGD Males, Korogocho, R2.**

That is why we are suggesting that after research, the results should be released earlier. For instance, there are very many cancer cases nowadays; if one is at stage 1, [it is preferable to begin] treatment early [rather than] waiting until stage 4, which requires millions [of shillings], which we do not have. So, it would be [beneficial] to receive the results [as soon as possible] so that [low-income] people can receive treatment. **FGD Males, Korogocho, R1**.

### Emotional reactions to returning results

The participants described mixed feelings on receiving their results. Some were pleased and said the results gave them hope while others feared the results*.*

When you give the results to the participants, it gives them hope because once they know their status and then they are told it is not dangerous and they will not have to incur unnecessary expenses. **FGD Males, Korogocho, R1**.

[It] is the fear of the unknown. It is like when you are told you will have the results of the exams out. You are the one who did that exam but you have the fear, "might I have failed, or could I be a victim of the situation? **IDI Community Leader 6, Viwandani.**

### Types of results to be returned

A majority of the participants advocated for individual results and some wanted both individual and aggregate results returned to the community.

I think both results are relevant to the community, aggregate and individual results. Aggregate [results] will generally tell the community how they are fairing; what is lacking in the community, what they need to improve on, it will be like an educative kind of result. **FGD Research Team, AWI-Gen I, R1.**

I prefer returning results to individual participants to avoid the stigma associated with such diseases. So returning results to individuals is the best way that is known between you and the doctor. **FGD Women, Korogocho, R7**.

Privacy was cited as a reason some preferred aggregate results so that the community members do not find out who was among those found with a certain condition. Some participants also misunderstood what it meant to return individual results. For some who preferred individual results, they misunderstood to think that individual results would be publicised to the community so they argued that results are specific to individuals and that it should not be returned to the community. They were concerned that information about their health status would become public due and they related it to the previous stigmatization of illnesses in the community.

When it comes to the [aggregate], in one way or the other, people will not know anyone’s [status] as people [will] not be able to tell whether who belong to the 70% or 20% [group]. **FGD Males, Korogocho, R1**.

Individual [results] are best because you will know what you are suffering from and address it at an individual level. For instance, those with HIV face a lot of stigmatization and you hear people saying that "leave that person alone he/she has HIV.”But, if you know it individually, then it's the best thing that can happen to you. **FGD Males, Korogocho, R5**.

[It] is okay [to provide results] provided the names of [those] afflicted is not made public. **IDI Community Leader 1, Korogocho.**

Some study participants noted that the aggregate results would be informative to the community but they also felt that it needed to be followed up with some community education in regards to what the next steps would be for those diagnosed with conditions.

[It] is okay provided the names of [those] afflicted is not made public. However, general percentages for individual diseases is okay as it enlightens the community members and it should be followed up by community education on what to do regarding their various diagnosis. **IDI Community Leader 1, Korogocho.**

### Methods of returning results

The community proposed several methods of returning results. Some suggested methods that would ensure confidentiality in returned results, such as using community health workers and mobile phones. Others proposed using community gatherings, religious institutions (Churches), chiefs, posters, and local newspapers.

Use phone numbers to reach us… You can also use community health workers [because] they know how to maintain the confidentiality of participants by coming to our place in person and finding us.…..Through the church, chief, all those you can find **FGD Women, Korogocho, R3.**

Via mobile phone because some people don’t like their affairs to be known. **FGD Males, Viwandani, R10.**

By using posters, through the phone…. **FGD Males, Korogocho, R2.**

…through newspaper reports **FGD REC, R3.**

### Timing of returning results

Study participants raised concerns about the time they received their results. The majority of the participants felt that returning results took long and proposed that the time be shortened in future research. Some want the results immediately while others felt that the results should be returned within one year.

It takes a very long time before those who participated receive feedback, so [this] should be addressed so that in the future, after the research, feedback is given to the participants immediately…... **FGD Males, Korogocho, R1.**

Participants believed that receiving results immediately or within a year of the research would help them make decisions based on the findings shared. They noted that with prompt results, they would seek medical attention promptly for the conditions they are diagnosed with. Others noted that their health status may deteriorate further if the results are delayed. Others said the late return of results makes the participants’ lose interest in the results and they forget what the study was about.

It would be good if you came [back within a] year. You know now how I was in the previous year is not the same way I will be this year. I will have suffered because this problem is spreading. **IDI AWI-Gen I Participant 2, Korogocho.**

…you know when someone gets tested, they are anxious to know their results promptly and it keeps on disturbing them where they ask when the results will be returned so that they can know their health status. That enthusiasm exists but when you delay returning the results, participants forget about them completely. When they forget and you return to explain [the results], they won't be able to remember what the test was all about.. **IDI Community Leader 7, Korogocho.**

They thought that a genetic discovery would encourage people to be more vigilant in their preventive practices, adopt healthier lifestyles or increase their chances of receiving better treatment for various diseases.

If they can produce the results [on] the same day, it would [be] better if they gave us the results before leaving. Like for HIV tests, you get tested and receive the results the same day. For most blood tests, you get the results the same day, so this should be the same so that if you have an issue, you can start addressing it early enough. Most diseases are detected due to blood tests, so I would request them to make the results available [on] the same day of the tests. Even if they don't offer you treatment, they can inform you of what to do to control it and even seek medical care. **FGD Males, Korogocho, R3.**

### Benefits of returning results

The study participants felt that returning research results would help them make better health decisions for example seeking medical care, changing their diet, etc.

Receiving the results is a good idea because then you will know your status and then seek medical care from anywhere… **FGD Males, Korogocho, R6**.

Cardiovascular diseases are lifestyle diseases while some of them are inherited. For the lifestyle, they will know how to live healthily and leave certain behaviours that predispose them to get risks of cardiovascular diseases like smoking, taking a lot of fats in their food especially saturated fats, exercise; walking around, jogging, cycling. **FGD Research Team, AWI-Gen I, R4.**

### Understanding results

Study participants' understanding of their results is key for making informed decisions.

So dissemination is a requirement that researchers must do and that is the challenge when you deal with complex subjects like genomics, to be able to bring it down to the level of the people's understanding…. **FGD REC, R1.**

Is good because when one's family gets to know that either their mother or sister has a particular condition, they will get her to the doctor and give the doctor a medical history so the doctor will know where to start from. **IDI AWI-Gen I participant 2, Viwandani**.

### Good ethical practices when returning results

When returning research results to study participants, researchers should uphold good ethical practices. The study participants expect the researchers to explain the results to them. This should be done in a private room and confidentiality should be maintained at all times. Those found with a problem also need to be counselled. In addition, in order to protect the identity of study participants, others felt that aggregated results should not contain identifiers.

The researchers should take their time to explain to us about the results. Then, as I said earlier, we should be invited as a group before we get individual results in a private room with the researcher. This was the problem and you need to do this so that you don't get that problem. **FGD Women, Viwandani, R4**.

There’s confidentiality, so if the results are being returned on one on one level –to an individual designated participant, it should be private and confidential. It should not pass through another person; it should be directed to the respondent. But then if they are aggregated, there should be no mentioning of the names of the participants who participated in the study. **FGD Research Team, AWI-Gen I, R4.**

Members of the research ethics committee felt that when disseminating results, the researchers needed to take precautions when disseminating sensational results such as ethnic groupings. The information should not be in a manner that will cause undue anxiety and panic among the study participants.

You will be able to identify ethnic groupings. You will need to disseminate this information in a way that the people will understand and I have said that it should be in such a way that does not cause any anxiety or panic among the people. **FGD REC, R5.**

Let them [to] be told properly, in a way that they will not feel [anxious]. Or there is something that you have thrown to the family that will cause anxiety. **IDI AWI-Gen I participant 1, Korogocho.**

### Challenges and solutions of feedbacking results

Given the complex nature of the medical reports, most participants will fiind it difficult to interpret them.

Challenges are sometimes in the interpretation of results. If medical reports are involved that need to be interpreted, it can be a challenge [as] not everyone can interpret medical results. **FGD Research Team, AWI-Gen I, R4.**

Some results may result in discrimination and stigmatization of the study participants especially if the results are not conveyed in the right way.

For example, suppose you discover a certain genome sequence of sickle cell anaemia in a particular community. When that gets out, as we have said it is important you learn how to package the results. [If not packaged well], it could result in stigma and discrimination issues **FGD REC, R6.**

If confidentiality and privacy are not adhered to, this can negatively affect the study participants and their family members. In some cases, it can result in violence among family members. Some family members may start dividing personal possessions among themselves.

[If] confidentiality and privacy are not adhered to, then it will negatively impact families and there will be those who will not receive the news well. Such kind of results can cause a lot of violence it is because of such kind of things [cause] shock. **IDI AWI-Gen I participant 2, Korogocho.**

There could be because you know when the results come out and they find out that you are sick, they start being sad, if you have wealth... they start dividing [personal possessions] yet you’re not dead. Such results are problematic. **IDI Community Leader 8, Korogocho.**

Some participants were in denial, especially when the results did not favour their expectations. Some went to the extent of seeking repeat tests in different health facilities to validate the results while others experienced fear of the unknown.

…..They would know their status but their stand is negative, they don't want to be affected. So those are the disadvantages of returning, because those who had a stand of negative results and it happens that they are positive, will be disturbed. They will be in denial. You can test them and even explain to them but they will refute it. They will say let me go elsewhere so that I can get tested and have assurance…. **IDI Community Leader 7, Korogocho**.

The strategies proposed by participants to address the challenges included increasing the research participants' and the general public's awareness regarding the feedback of results.

The best way would be first to sensitize the participants who participated in the research study…. Inform them that [return of results took] long because the samples were not being examined [locally], they were being tested outside the country. **IDI Community Leader 1, Korogocho.**

## Discussion

This study sought to explore stakeholders’ perspectives on returning genomic results at the participant and community level in two Nairobi informal settlements of Kenya. Findings were generally positive towards the return of results though cited challenges were regarding how the return of results needs to be done. The positives regarding the return of results were 1) it motivates participation in future research studies, 2) it is a sign of respect to study participants, and 3) study participants will know their health status and seek appropriate care. The major challenges with returning participants' results were the complex nature of medical reports, discrimination, stigmatization, denial of results, and lack of confidentiality and privacy. These findings contribute to the evidence on whether or not to return research results to research participants and their communities. The findings will also inform the design and conduct of similar studies on the return of results.

Returning research findings provides value to the participants, community, and scientific stakeholders. Participants reported a strong preference for receiving research findings in which they had participated in and have relevance to their health. The study findings are consistent with prior studies where participants want to receive findings that are significant to their health or that of a loved one or are actionable [5, 16, 43–50] as this increases transparency in research and demonstrates researchers’ respect for their participation [16, 51]. This also builds trust between researchers and study participants. Researchers do not generally return research findings at the participant and community level [2, 5, 16, 45, 52]. This has been witnessed in studies where researchers enter the communities, collect samples and data and then leave without ever returning to their study sites to inform participants of their findings leaving the relationship between researchers and the communities strained [53]. Currently, most research ethics committees (RECs) institutions require researchers to engage the communities before commencing and upon completion of studies [23, 28, 54–58]. Due to the variability of research results and the lack of reference information, participants should be allowed to choose which results to receive during the consenting process [59, 60].

The return of results involves various types of data collected using various methods tailored to the nature of the study. Research results are associated with a greater degree of uncertainties at individual results compared to aggregated results due to incomplete scientific knowledge [61, 62]. In this study, some participants expressed a desire to receive individual results while others indicated their participation contributed to results and did not expect to receive individual results. This contrasts with several studies' findings where most study participants favoured access to individual results even with no known clinical significance [44, 63]. However, there are concerns surrounding the return of research findings due to the possibility of a negative impact on the participant. In some cases, participants cited individual results as a burden as they may cause emotional or other distress, which is consistent with previous reports [22, 64]. Generally, adaptation to this information by participants may motivate them to take the initiative to reduce health risks [65–67].

Different communication modalities may be appropriate in different contexts [68]. A variety of methods are used to returning research results in studies with a genetic basis of human diseases [69]. In-person discussions, phone or video-conference based discussions, electronic delivery, and mailing of printed materials are all common methods of communicating results [68]. Methods proposed to be used by researchers to return results were through telephone, via mail, in person, and via referral to a physician. The choice of communication modalities of research results should be based on the results' significance, risk level, and the available types of intervention [16, 54].

Respondents from the current study stated that researchers need to consider communicating research results appropriately for participants with varying needs, resources, and backgrounds. This finding is consistent with prior studies where respondents recommended the researchers provide context for the results they provide [70, 71]. This requires skillful communication and improved language reduces potential misunderstandings between the result conveyor and the study participant [72–74]. Researchers should frequently use a genetic counsellor or a trained professional to communicate test results effectively and accurately [3, 75, 76]. They have a rich source of expertise and experience in explaining complex information to individuals tailored to respect their cultural, religious, and ethnic beliefs [77–79].

Given the complexity and uncertainty in genetic-related risk, it is difficult to communicate genomic results particularly when it relates to something already problematic in nature [61]. The value of genomic results to study participants can be either clinical or personal. Results that are clinically actionable to guide preventive interventions or treatment should be returned. Presentation of genetic test results on individual characteristics may need greater tailoring especially when results are not delivered in a clinical setting or by a trained healthcare professional [80].

## Conclusion

Although the return of individual results is not currently common in research studies, some investigators are already returning research results to individual participants [68]. The results in this study also highlight that there is little published on the return of research results to individuals and communities thus research gaps remain that would better inform the development of best practices and guidance for other types of research results. Research teams would benefit from guidance on accomplishing the challenging task of accurately communicating both the individual level and aggregate research results. Researchers need to effectively enable participants to understand individual results and how to use individual results. They should also caution against the overuse of individual results.

## Data Availability

The datasets generated and/or analysed during the current study are not publicly available due limitations of ethical approval involving participants data and anonymity but are available from the corresponding author on reasonable request.

## Abbreviations

AWI-Gen: African Wits-International Network for Demographic Evaluation of Population and Their Health (INDEPTH) partnership for Genomic Studies
CHV: Community Health Volunteer
COREQ: Criteria for Reporting Qualitative
FGDs: Focus group discussions
H3Africa: Human Heredity and Health in Africa
IDIs: In-depth Interviews
NUHDSS: Nairobi Urban Health and Demographic Surveillance System
REC: Research Ethics Committee
